# Tetra­kis(μ-2,4-difluoro­benzoato)bis­[(2,4-difluoro­benzoato)(1,10-phenanthroline)gadolinium(III)]

**DOI:** 10.1107/S1600536808004431

**Published:** 2008-03-05

**Authors:** Shou-Bin Wang, Hong-Mei He, Sheng Li, Kun Tang

**Affiliations:** a College of Chemistry and Chemical Engineering, Henan University, Kaifeng 475003, People’s Republic of China; b College of Medicine, Henan University, Kaifeng 475003, People’s Republic of China

## Abstract

In the title compound, [Gd_2_(C_7_H_3_F_2_O_2_)_6_(C_12_H_8_N_2_)_2_], the asymmetric unit comprises one Gd^3+^ cation chelated by two 2,4-difluoro­benzoate and one 1,10-phenanthroline ligands. Two cations are linked into a centrosymmetric dimer via three bridging carboxyl­ate groups of 2,4-difluoro­benzoate ligands. Each Gd^3+^ ion is nine-coordinated by seven O atoms and two N atoms.

## Related literature

For related literature, see: Church & Halvorson (1959 ▶); Chung *et al.* (1971 ▶); Okabe & Oya (2000 ▶); Serre *et al.* (2005 ▶); Pocker & Fong (1980 ▶); Scapin *et al.* (1997 ▶).
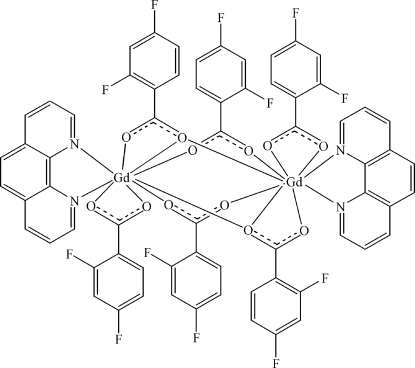


## Experimental

### 

#### Crystal data

[Gd_2_(C_7_H_3_F_2_O_2_)_6_(C_12_H_8_N_2_)_2_]
*M* *_r_* = 1617.47
Monoclinic, 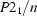

*a* = 15.132 (3) Å
*b* = 13.663 (3) Å
*c* = 15.286 (3) Å
β = 109.364 (2)°
*V* = 2981.6 (9) Å^3^
*Z* = 2
Mo *K*α radiation
μ = 2.31 mm^−1^
*T* = 295 (2) K
0.33 × 0.14 × 0.08 mm

#### Data collection

Bruker APEXII CCD area-detector diffractometer
Absorption correction: multi-scan (*SADABS*; Bruker, 2001 ▶) *T*_min_ = 0.516, *T*_max_ = 0.837
15634 measured reflections
5535 independent reflections
4598 reflections with *I* > 2σ(*I*)
*R*_int_ = 0.037

#### Refinement

*R*[*F* ^2^ > 2σ(*F* ^2^)] = 0.032
*wR*(*F* ^2^) = 0.095
*S* = 1.00
5535 reflections
433 parameters
H-atom parameters constrained
Δρ_max_ = 1.69 e Å^−3^
Δρ_min_ = −0.64 e Å^−3^

### 

Data collection: *APEX2* (Bruker, 2004 ▶); cell refinement: *SAINT-Plus* (Bruker, 2001 ▶); data reduction: *SAINT-Plus*; program(s) used to solve structure: *SHELXS97* (Sheldrick, 2008 ▶); program(s) used to refine structure: *SHELXL97* (Sheldrick, 2008 ▶); molecular graphics: *SHELXTL* (Sheldrick, 2008 ▶); software used to prepare material for publication: *SHELXTL*.

## Supplementary Material

Crystal structure: contains datablocks I, global. DOI: 10.1107/S1600536808004431/cf2184sup1.cif

Structure factors: contains datablocks I. DOI: 10.1107/S1600536808004431/cf2184Isup2.hkl

Additional supplementary materials:  crystallographic information; 3D view; checkCIF report

## Figures and Tables

**Fig. 1.**
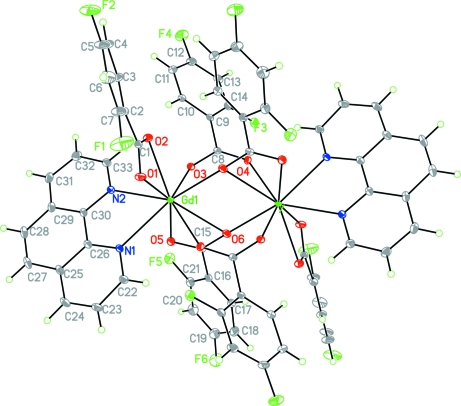
The molecular structure of (I), showing 30% probability displacement ellipsoids. Unlabeled atoms are at the symmetry position (-x + 2, -y, -z + 2).

## supplementary crystallographic information

### Comment

In recent years, carboxylates have been widely used as polydentate ligands, which can coordinate to transition or rare earth ions yielding complexes with interesting properties that are useful in materials science (Church & Halvorson, 1959; Chung *et al.*, 1971) and in biological systems (Okabe & Oya, 2000; Serre *et al.*, 2005; Pocker & Fong, 1980; Scapin *et al.*, 1997). Here we report the synthesis and X-ray crystal structure analysis of the title compound.

The molecular structure is shown in Fig. 1. Gd(III) is chelated by two 2,4-difluorobenzoate and one 1,10-phenanthroline ligands. Two cations are linked into a dimer *via* three bridging carboxylate groups of 2,4-difluorobenzoate ligands. Each Gd(III) ion is nine-coordinated by seven O atoms and two N atoms.

### Experimental

A mixture of gadolinium(III) chloride (0.5 mmol), 2,4-difluorobenzoic acid (1 mmol), sodium hydroxide (1 mmol), 1,10-phenanthroline (0.5 mmol), water (8 ml) and ethanol (8 ml) in a 25 ml Teflon-lined stainless steel autoclave was kept at 433 K for three days. Colorless crystals were obtained after cooling to room temperature, with a yield of 16%. Anal. Calc. for C_33_H_17_F_6_GdN_2_O_6_: C 48.98, H 2.10, N 3.46%; Found: C 48.88, H 2.12, N 3.98%.

### Refinement

All H atoms were positioned geometrically and refined as riding atoms with C—H = 0.93 Å and *U*_iso_(H) = 1.2*U*_eq_(C).

### Crystal data

**Table:**

[Gd_2_(C_7_H_3_F_2_O_2_)_6_(C_12_H_8_N_2_)_2_]	*F*_000_ = 1580
*M**_r_* = 1617.47	*D*_x_ = 1.802 Mg m^−^^3^
Monoclinic, *P*2_1_/*n*	Mo *K*α radiation λ = 0.71073 Å
Hall symbol: -P 2yn	Cell parameters from 5535 reflections
*a* = 15.132 (3) Å	θ = 2.1–25.5º
*b* = 13.663 (3) Å	µ = 2.31 mm^−^^1^
*c* = 15.286 (3) Å	*T* = 295 (2) K
β = 109.364 (2)º	Block, colourless
*V* = 2981.6 (9) Å^3^	0.33 × 0.14 × 0.08 mm
*Z* = 2	

### Data collection

**Table:**

Bruker APEXII CCD area-detector diffractometer	5535 independent reflections
Radiation source: fine-focus sealed tube	4598 reflections with *I* > 2σ(*I*)
Monochromator: graphite	*R*_int_ = 0.037
*T* = 295(2) K	θ_max_ = 25.5º
φ and ω scans	θ_min_ = 2.1º
Absorption correction: multi-scan(SADABS; Bruker, 2001)	*h* = −14→18
*T*_min_ = 0.516, *T*_max_ = 0.837	*k* = −16→16
15634 measured reflections	*l* = −17→18

### Refinement

**Table:**

Refinement on *F*^2^	Secondary atom site location: difference Fourier map
Least-squares matrix: full	Hydrogen site location: inferred from neighbouring sites
*R*[*F*^2^ > 2σ(*F*^2^)] = 0.032	H-atom parameters constrained
*wR*(*F*^2^) = 0.095	*w* = 1/[σ^2^(*F*_o_^2^) + (0.0528*P*)^2^ + 4.39*P*] where *P* = (*F*_o_^2^ + 2*F*_c_^2^)/3
*S* = 1.00	(Δ/σ)_max_ = 0.008
5535 reflections	Δρ_max_ = 1.69 e Å^−^^3^
433 parameters	Δρ_min_ = −0.64 e Å^−^^3^
Primary atom site location: structure-invariant direct methods	Extinction correction: none

### Special details

**Table:**

Geometry. All e.s.d.'s (except the e.s.d. in the dihedral angle between two l.s. planes) are estimated using the full covariance matrix. The cell e.s.d.'s are taken into account individually in the estimation of e.s.d.'s in distances, angles and torsion angles; correlations between e.s.d.'s in cell parameters are only used when they are defined by crystal symmetry. An approximate (isotropic) treatment of cell e.s.d.'s is used for estimating e.s.d.'s involving l.s. planes.
Refinement. Refinement of *F*^2^ against ALL reflections. The weighted *R*-factor *wR* and goodness of fit *S* are based on *F*^2^, conventional *R*-factors *R* are based on *F*, with *F* set to zero for negative *F*^2^. The threshold expression of *F*^2^ > σ(*F*^2^) is used only for calculating *R*-factors(gt) *etc*. and is not relevant to the choice of reflections for refinement. *R*-factors based on *F*^2^ are statistically about twice as large as those based on *F*, and *R*- factors based on ALL data will be even larger.

### Fractional atomic coordinates and isotropic or equivalent isotropic displacement parameters (Å^2^)

**Table:**

	*x*	*y*	*z*	*U*_iso_*/*U*_eq_	
Gd1	0.997885 (14)	0.144628 (14)	0.957975 (13)	0.02862 (9)	
C1	1.1346 (3)	0.2166 (3)	0.8849 (3)	0.0373 (10)	
C2	1.2110 (3)	0.2446 (3)	0.8476 (3)	0.0428 (11)	
C3	1.1982 (4)	0.2280 (4)	0.7539 (4)	0.0530 (13)	
H3	1.1411	0.2041	0.7152	0.064*	
C4	1.2694 (5)	0.2467 (5)	0.7181 (4)	0.0725 (19)	
H4	1.2606	0.2353	0.6558	0.087*	
C5	1.3513 (5)	0.2816 (5)	0.7747 (5)	0.076 (2)	
C6	1.3667 (5)	0.2995 (6)	0.8637 (6)	0.095 (3)	
H6	1.4243	0.3240	0.9009	0.113*	
C7	1.2965 (4)	0.2815 (5)	0.9009 (4)	0.0667 (17)	
C8	0.9247 (3)	−0.0549 (3)	0.8221 (3)	0.0316 (9)	
C9	0.9086 (3)	−0.0732 (3)	0.7207 (3)	0.0328 (9)	
C10	0.9380 (3)	−0.0029 (3)	0.6705 (3)	0.0413 (11)	
H10	0.9713	0.0512	0.7013	0.050*	
C11	0.9189 (4)	−0.0117 (4)	0.5764 (3)	0.0573 (14)	
H11	0.9399	0.0351	0.5437	0.069*	
C12	0.8686 (5)	−0.0904 (5)	0.5324 (3)	0.0656 (17)	
C13	0.8377 (4)	−0.1626 (4)	0.5768 (3)	0.0541 (14)	
H13	0.8030	−0.2153	0.5446	0.065*	
C14	0.8605 (3)	−0.1536 (3)	0.6721 (3)	0.0407 (11)	
C15	0.8390 (3)	0.0592 (3)	1.0120 (3)	0.0344 (9)	
C16	0.7585 (3)	0.0448 (3)	1.0482 (3)	0.0422 (11)	
C17	0.7753 (5)	0.0095 (4)	1.1374 (4)	0.0631 (16)	
H17	0.8347	−0.0130	1.1717	0.076*	
C18	0.7040 (6)	0.0075 (5)	1.1760 (5)	0.085 (2)	
H18	0.7150	−0.0182	1.2350	0.102*	
C19	0.6197 (6)	0.0427 (6)	1.1277 (6)	0.092 (3)	
C20	0.5978 (4)	0.0755 (5)	1.0382 (5)	0.0761 (19)	
H20	0.5378	0.0968	1.0048	0.091*	
C21	0.6685 (4)	0.0755 (4)	0.9998 (4)	0.0532 (13)	
C22	0.9877 (4)	0.3346 (4)	1.1111 (3)	0.0454 (12)	
H22	1.0244	0.2895	1.1532	0.054*	
C23	0.9628 (4)	0.4212 (4)	1.1453 (4)	0.0553 (14)	
H23	0.9809	0.4318	1.2089	0.066*	
C24	0.9121 (4)	0.4896 (4)	1.0857 (4)	0.0548 (14)	
H24	0.8961	0.5479	1.1082	0.066*	
C25	0.8838 (3)	0.4726 (3)	0.9899 (4)	0.0440 (11)	
C26	0.9099 (3)	0.3819 (3)	0.9608 (3)	0.0371 (10)	
C27	0.8324 (4)	0.5425 (4)	0.9223 (4)	0.0569 (15)	
H27	0.8156	0.6020	0.9418	0.068*	
C28	0.8081 (4)	0.5232 (4)	0.8309 (4)	0.0547 (14)	
H28	0.7764	0.5704	0.7882	0.066*	
C29	0.8303 (3)	0.4316 (3)	0.7986 (4)	0.0452 (11)	
C30	0.8798 (3)	0.3602 (3)	0.8629 (3)	0.0361 (10)	
C31	0.8017 (4)	0.4072 (4)	0.7045 (4)	0.0551 (14)	
H31	0.7696	0.4526	0.6600	0.066*	
C32	0.8213 (4)	0.3157 (5)	0.6782 (4)	0.0598 (15)	
H32	0.8018	0.2980	0.6160	0.072*	
C33	0.8708 (4)	0.2504 (4)	0.7466 (3)	0.0480 (12)	
H33	0.8836	0.1887	0.7281	0.058*	
F1	1.3151 (4)	0.3046 (6)	0.9873 (4)	0.153 (3)	
F2	1.4208 (3)	0.3002 (4)	0.7394 (4)	0.133 (2)	
F3	0.8319 (2)	−0.2247 (2)	0.71639 (19)	0.0520 (7)	
F4	0.8467 (4)	−0.0971 (4)	0.4386 (2)	0.1107 (16)	
F5	0.6472 (2)	0.1067 (3)	0.9116 (2)	0.0688 (9)	
F6	0.5496 (4)	0.0443 (4)	1.1648 (4)	0.141 (2)	
N1	0.9615 (2)	0.3134 (3)	1.0209 (2)	0.0331 (8)	
N2	0.9010 (3)	0.2706 (3)	0.8367 (2)	0.0355 (8)	
O1	1.1385 (2)	0.2404 (3)	0.9662 (2)	0.0486 (8)	
O2	1.0671 (2)	0.1674 (2)	0.8334 (2)	0.0418 (7)	
O3	0.9197 (2)	0.0335 (2)	0.84238 (19)	0.0360 (7)	
O4	0.9422 (2)	−0.1250 (2)	0.8774 (2)	0.0398 (7)	
O5	0.8436 (2)	0.1391 (2)	0.9743 (2)	0.0440 (8)	
O6	0.8994 (2)	−0.0069 (2)	1.0241 (2)	0.0449 (8)	

### Atomic displacement parameters (Å^2^)

**Table:**

	*U*^11^	*U*^22^	*U*^33^	*U*^12^	*U*^13^	*U*^23^
Gd1	0.03159 (14)	0.02662 (13)	0.02902 (13)	−0.00158 (8)	0.01190 (9)	−0.00349 (8)
C1	0.043 (3)	0.030 (2)	0.045 (3)	−0.0028 (19)	0.022 (2)	−0.0029 (19)
C2	0.046 (3)	0.036 (3)	0.055 (3)	−0.009 (2)	0.029 (2)	−0.006 (2)
C3	0.060 (3)	0.056 (3)	0.054 (3)	−0.016 (3)	0.034 (3)	−0.004 (2)
C4	0.087 (5)	0.082 (5)	0.071 (4)	−0.032 (4)	0.056 (4)	−0.020 (3)
C5	0.075 (4)	0.088 (5)	0.094 (5)	−0.041 (4)	0.065 (4)	−0.042 (4)
C6	0.060 (4)	0.118 (7)	0.118 (6)	−0.048 (4)	0.047 (4)	−0.043 (5)
C7	0.062 (4)	0.089 (5)	0.058 (3)	−0.031 (3)	0.031 (3)	−0.033 (3)
C8	0.028 (2)	0.033 (2)	0.034 (2)	−0.0040 (17)	0.0107 (17)	−0.0075 (18)
C9	0.034 (2)	0.034 (2)	0.028 (2)	0.0058 (18)	0.0075 (17)	−0.0058 (17)
C10	0.049 (3)	0.039 (3)	0.039 (2)	0.005 (2)	0.020 (2)	−0.002 (2)
C11	0.081 (4)	0.058 (3)	0.042 (3)	0.010 (3)	0.032 (3)	0.006 (3)
C12	0.095 (5)	0.072 (4)	0.031 (3)	0.014 (3)	0.021 (3)	−0.009 (3)
C13	0.067 (4)	0.054 (3)	0.034 (3)	0.004 (3)	0.006 (2)	−0.019 (2)
C14	0.046 (3)	0.035 (3)	0.038 (2)	0.008 (2)	0.011 (2)	−0.0043 (19)
C15	0.031 (2)	0.040 (2)	0.033 (2)	0.0016 (19)	0.0113 (18)	−0.0068 (19)
C16	0.047 (3)	0.031 (2)	0.057 (3)	−0.001 (2)	0.029 (2)	−0.001 (2)
C17	0.089 (5)	0.047 (3)	0.071 (4)	0.018 (3)	0.051 (3)	0.011 (3)
C18	0.117 (6)	0.069 (4)	0.103 (5)	0.022 (4)	0.081 (5)	0.028 (4)
C19	0.104 (6)	0.080 (5)	0.134 (7)	0.002 (4)	0.095 (6)	0.020 (5)
C20	0.049 (3)	0.073 (4)	0.120 (6)	−0.006 (3)	0.047 (4)	0.006 (4)
C21	0.044 (3)	0.044 (3)	0.079 (4)	−0.006 (2)	0.030 (3)	0.005 (3)
C22	0.053 (3)	0.041 (3)	0.045 (3)	−0.003 (2)	0.020 (2)	−0.010 (2)
C23	0.065 (3)	0.055 (3)	0.056 (3)	−0.012 (3)	0.033 (3)	−0.024 (3)
C24	0.057 (3)	0.038 (3)	0.078 (4)	−0.004 (2)	0.034 (3)	−0.023 (3)
C25	0.035 (2)	0.031 (2)	0.070 (3)	−0.0048 (19)	0.023 (2)	−0.010 (2)
C26	0.030 (2)	0.032 (2)	0.053 (3)	−0.0045 (18)	0.018 (2)	−0.006 (2)
C27	0.044 (3)	0.029 (3)	0.095 (5)	0.004 (2)	0.019 (3)	−0.006 (3)
C28	0.040 (3)	0.036 (3)	0.082 (4)	0.003 (2)	0.012 (3)	0.011 (3)
C29	0.035 (2)	0.037 (3)	0.062 (3)	0.001 (2)	0.014 (2)	0.010 (2)
C30	0.030 (2)	0.031 (2)	0.049 (3)	−0.0040 (17)	0.015 (2)	−0.0011 (19)
C31	0.049 (3)	0.054 (3)	0.059 (3)	0.007 (3)	0.015 (3)	0.023 (3)
C32	0.062 (4)	0.073 (4)	0.039 (3)	0.008 (3)	0.010 (3)	0.009 (3)
C33	0.055 (3)	0.051 (3)	0.037 (3)	0.010 (2)	0.013 (2)	0.000 (2)
F1	0.123 (4)	0.247 (7)	0.102 (4)	−0.105 (5)	0.056 (3)	−0.058 (4)
F2	0.114 (4)	0.163 (5)	0.174 (5)	−0.081 (4)	0.116 (4)	−0.077 (4)
F3	0.0626 (18)	0.0388 (15)	0.0505 (16)	−0.0103 (13)	0.0131 (14)	−0.0080 (13)
F4	0.176 (5)	0.118 (4)	0.0343 (18)	−0.005 (3)	0.030 (2)	−0.014 (2)
F5	0.0458 (18)	0.080 (2)	0.076 (2)	0.0011 (17)	0.0149 (17)	0.0107 (19)
F6	0.130 (4)	0.150 (5)	0.206 (6)	0.017 (3)	0.141 (4)	0.043 (4)
N1	0.0321 (19)	0.0286 (18)	0.042 (2)	0.0002 (15)	0.0168 (16)	−0.0034 (16)
N2	0.038 (2)	0.033 (2)	0.0369 (19)	0.0011 (16)	0.0140 (16)	−0.0016 (16)
O1	0.053 (2)	0.054 (2)	0.0486 (19)	−0.0209 (17)	0.0298 (17)	−0.0205 (16)
O2	0.0395 (18)	0.0489 (19)	0.0399 (17)	−0.0076 (15)	0.0170 (15)	−0.0039 (15)
O3	0.0447 (18)	0.0327 (16)	0.0306 (15)	−0.0033 (13)	0.0124 (13)	−0.0070 (12)
O4	0.057 (2)	0.0307 (16)	0.0282 (15)	−0.0037 (14)	0.0100 (14)	−0.0012 (13)
O5	0.0467 (19)	0.0359 (18)	0.057 (2)	−0.0036 (14)	0.0269 (17)	0.0006 (15)
O6	0.0462 (19)	0.049 (2)	0.0405 (18)	0.0153 (16)	0.0155 (15)	0.0000 (15)

### Geometric parameters (Å, °)

**Table:**

Gd1—O3	2.332 (3)	C15—C16	1.509 (6)
Gd1—O4^i^	2.391 (3)	C16—C21	1.382 (7)
Gd1—O6^i^	2.397 (3)	C16—C17	1.388 (7)
Gd1—O5	2.429 (3)	C17—C18	1.391 (9)
Gd1—O1	2.465 (3)	C17—H17	0.930
Gd1—O2	2.479 (3)	C18—C19	1.334 (11)
Gd1—N2	2.597 (4)	C18—H18	0.930
Gd1—N1	2.627 (3)	C19—F6	1.359 (7)
Gd1—O6	2.921 (4)	C19—C20	1.372 (10)
C1—O2	1.257 (5)	C20—C21	1.381 (8)
C1—O1	1.267 (5)	C20—H20	0.930
C1—C2	1.499 (6)	C21—F5	1.346 (6)
C2—C7	1.376 (7)	C22—N1	1.334 (6)
C2—C3	1.398 (7)	C22—C23	1.395 (7)
C3—C4	1.385 (7)	C22—H22	0.930
C3—H3	0.930	C23—C24	1.353 (8)
C4—C5	1.342 (9)	C23—H23	0.930
C4—H4	0.930	C24—C25	1.402 (7)
C5—C6	1.325 (10)	C24—H24	0.930
C5—F2	1.355 (6)	C25—C26	1.416 (6)
C6—C7	1.383 (9)	C25—C27	1.431 (7)
C6—H6	0.930	C26—N1	1.362 (6)
C7—F1	1.295 (7)	C26—C30	1.444 (7)
C8—O4	1.246 (5)	C27—C28	1.348 (8)
C8—O3	1.256 (5)	C27—H27	0.930
C8—C9	1.507 (5)	C28—C29	1.426 (7)
C9—C14	1.390 (6)	C28—H28	0.930
C9—C10	1.391 (6)	C29—C31	1.398 (8)
C10—C11	1.376 (6)	C29—C30	1.411 (6)
C10—H10	0.930	C30—N2	1.358 (5)
C11—C12	1.360 (8)	C31—C32	1.375 (8)
C11—H11	0.930	C31—H31	0.930
C12—C13	1.364 (9)	C32—C33	1.390 (7)
C12—F4	1.364 (6)	C32—H32	0.930
C13—C14	1.387 (7)	C33—N2	1.329 (6)
C13—H13	0.930	C33—H33	0.930
C14—F3	1.335 (6)	O4—Gd1^i^	2.391 (3)
C15—O5	1.247 (5)	O6—Gd1^i^	2.397 (3)
C15—O6	1.255 (5)		
			
O3—Gd1—O4^i^	130.13 (10)	F3—C14—C13	117.3 (4)
O3—Gd1—O6^i^	73.87 (11)	F3—C14—C9	120.3 (4)
O4^i^—Gd1—O6^i^	77.68 (10)	C13—C14—C9	122.4 (5)
O3—Gd1—O5	78.14 (11)	O5—C15—O6	123.2 (4)
O4^i^—Gd1—O5	85.99 (12)	O5—C15—C16	117.4 (4)
O6^i^—Gd1—O5	124.91 (11)	O6—C15—C16	119.3 (4)
O3—Gd1—O1	126.62 (10)	C21—C16—C17	117.4 (5)
O4^i^—Gd1—O1	89.20 (11)	C21—C16—C15	122.3 (4)
O6^i^—Gd1—O1	83.97 (12)	C17—C16—C15	119.9 (5)
O5—Gd1—O1	148.60 (11)	C16—C17—C18	120.6 (6)
O3—Gd1—O2	74.75 (10)	C16—C17—H17	119.7
O4^i^—Gd1—O2	135.54 (11)	C18—C17—H17	119.7
O6^i^—Gd1—O2	76.31 (11)	C19—C18—C17	119.5 (6)
O5—Gd1—O2	138.42 (11)	C19—C18—H18	120.3
O1—Gd1—O2	52.79 (10)	C17—C18—H18	120.3
O3—Gd1—N2	82.32 (11)	C18—C19—F6	120.7 (7)
O4^i^—Gd1—N2	138.85 (11)	C18—C19—C20	122.7 (6)
O6^i^—Gd1—N2	142.42 (11)	F6—C19—C20	116.5 (8)
O5—Gd1—N2	76.13 (11)	C19—C20—C21	117.4 (6)
O1—Gd1—N2	87.58 (12)	C19—C20—H20	121.3
O2—Gd1—N2	69.53 (11)	C21—C20—H20	121.3
O3—Gd1—N1	137.20 (11)	F5—C21—C20	117.7 (5)
O4^i^—Gd1—N1	76.43 (11)	F5—C21—C16	119.9 (5)
O6^i^—Gd1—N1	148.65 (11)	C20—C21—C16	122.4 (6)
O5—Gd1—N1	70.40 (11)	N1—C22—C23	123.1 (5)
O1—Gd1—N1	78.31 (11)	N1—C22—H22	118.4
O2—Gd1—N1	111.36 (11)	C23—C22—H22	118.4
N2—Gd1—N1	62.75 (11)	C24—C23—C22	119.8 (5)
O3—Gd1—O6	67.18 (9)	C24—C23—H23	120.1
O4^i^—Gd1—O6	67.33 (10)	C22—C23—H23	120.1
O6^i^—Gd1—O6	77.87 (11)	C23—C24—C25	119.9 (5)
O5—Gd1—O6	47.51 (10)	C23—C24—H24	120.1
O1—Gd1—O6	152.79 (11)	C25—C24—H24	120.1
O2—Gd1—O6	138.53 (10)	C24—C25—C26	116.9 (5)
N2—Gd1—O6	119.04 (10)	C24—C25—C27	123.2 (5)
N1—Gd1—O6	107.62 (10)	C26—C25—C27	119.8 (5)
O2—C1—O1	121.1 (4)	N1—C26—C25	123.1 (4)
O2—C1—C2	117.8 (4)	N1—C26—C30	118.1 (4)
O1—C1—C2	121.1 (4)	C25—C26—C30	118.8 (4)
C7—C2—C3	116.7 (5)	C28—C27—C25	121.0 (5)
C7—C2—C1	124.1 (4)	C28—C27—H27	119.5
C3—C2—C1	119.1 (4)	C25—C27—H27	119.5
C4—C3—C2	120.9 (5)	C27—C28—C29	121.0 (5)
C4—C3—H3	119.6	C27—C28—H28	119.5
C2—C3—H3	119.6	C29—C28—H28	119.5
C5—C4—C3	119.0 (5)	C31—C29—C30	117.6 (5)
C5—C4—H4	120.5	C31—C29—C28	122.5 (5)
C3—C4—H4	120.5	C30—C29—C28	119.9 (5)
C6—C5—C4	122.6 (6)	N2—C30—C29	122.7 (4)
C6—C5—F2	118.5 (6)	N2—C30—C26	117.8 (4)
C4—C5—F2	118.9 (6)	C29—C30—C26	119.4 (4)
C5—C6—C7	119.4 (6)	C32—C31—C29	119.7 (5)
C5—C6—H6	120.3	C32—C31—H31	120.2
C7—C6—H6	120.3	C29—C31—H31	120.2
F1—C7—C2	122.5 (5)	C31—C32—C33	118.6 (5)
F1—C7—C6	116.0 (6)	C31—C32—H32	120.7
C2—C7—C6	121.4 (5)	C33—C32—H32	120.7
O4—C8—O3	126.0 (4)	N2—C33—C32	124.1 (5)
O4—C8—C9	119.7 (4)	N2—C33—H33	117.9
O3—C8—C9	114.2 (4)	C32—C33—H33	117.9
C14—C9—C10	117.2 (4)	C22—N1—C26	117.1 (4)
C14—C9—C8	123.9 (4)	C22—N1—Gd1	122.8 (3)
C10—C9—C8	118.7 (4)	C26—N1—Gd1	119.9 (3)
C11—C10—C9	121.5 (5)	C33—N2—C30	117.3 (4)
C11—C10—H10	119.3	C33—N2—Gd1	121.4 (3)
C9—C10—H10	119.3	C30—N2—Gd1	121.3 (3)
C12—C11—C10	118.3 (5)	C1—O1—Gd1	93.1 (3)
C12—C11—H11	120.8	C1—O2—Gd1	92.7 (3)
C10—C11—H11	120.8	C8—O3—Gd1	139.2 (3)
C11—C12—C13	123.7 (5)	C8—O4—Gd1^i^	136.2 (3)
C11—C12—F4	118.3 (6)	C15—O5—Gd1	106.2 (3)
C13—C12—F4	118.1 (6)	C15—O6—Gd1^i^	174.2 (3)
C12—C13—C14	116.8 (5)	C15—O6—Gd1	82.5 (3)
C12—C13—H13	121.6	Gd1^i^—O6—Gd1	102.13 (11)
C14—C13—H13	121.6		

Symmetry codes: (i) −*x*+2, −*y*, −*z*+2.

## References

[bb1] Bruker (2001). *SAINT-Plus* Bruker AXS Inc., Madison, Wisconsin, USA.

[bb2] Bruker (2004). *APEX2* Bruker AXS Inc., Madison, Wisconsin, USA.

[bb3] Chung, L., Rajan, K. S., Merdinger, E. & Crecz, N. (1971). *Biophys. J.* **11**, 469–475. 10.1016/S0006-3495(71)86229-X PMC1484009 5569493

[bb4] Church, B. S. & Halvorson, H. (1959). *Nature (London)*, **183**, 124–125. 10.1038/183124a0 13622720

[bb5] Okabe, N. & Oya, N. (2000). *Acta Cryst.* C**56**, 1416–1417. 10.1107/s0108270100012786 11118970

[bb6] Pocker, Y. & Fong, C. T. O. (1980). *Biochemistry*, **19**, 2045–2049. 10.1021/bi00551a006 6769470

[bb7] Scapin, G., Reddy, S. G., Zheng, R. & Blanchard, J. S. (1997). *Biochemistry*, **36**, 15081–15088. 10.1021/bi9719915 9398235

[bb8] Serre, C., Marrot, J. & Ferey, G. (2005). *Inorg. Chem.* **44**, 654–658. 10.1021/ic0487373 15679398

[bb9] Sheldrick, G. M. (2008). *Acta Cryst.* A**64**, 112–122. 10.1107/S0108767307043930 18156677

